# Evaluative contexts facilitate implicit mentalizing: relation to the broader autism phenotype and mental health

**DOI:** 10.1038/s41598-024-55075-9

**Published:** 2024-02-26

**Authors:** Ruihan Wu, Karen Leow, Nicole Yu, Ciara Rafter, Katia Rosenbaum, Antonia F. de C. Hamilton, Sarah J. White

**Affiliations:** 1 Institute of Cognitive Neuroscience, University College London, Alexandra House, 17 Queen Square, London, WC1N 3AZ UK; 2 National Institute of Education, Nanyang Technological University, Singapore, Singapore; 3 National University of Singapore, Singapore, Singapore; 4 Department for Environment, Food, and Rural Affairs, Manchester, UK

**Keywords:** Human behaviour, Social behaviour, Autism spectrum disorders

## Abstract

One promising account for autism is implicit mentalizing difficulties. However, this account and even the existence of implicit mentalizing have been challenged because the replication results are mixed. Those unsuccessful replications may be due to the task contexts not being sufficiently evaluative. Therefore, the current study developed a more evaluative paradigm by implementing a prompt question. This was assessed in 60 non-autistic adults and compared with a non-prompt version. Additionally, parents of autistic children are thought to show a genetic liability to autistic traits and cognition and often report mental health problems, but the broader autism phenotype (BAP) is an under-researched area. Thus, we also aimed to compare 33 BAP and 26 non-BAP mothers on mentalizing abilities, autistic traits, compensation and mental health. Our results revealed that more evaluative contexts can facilitate implicit mentalizing in BAP and non-BAP populations, and thus improve task reliability and replicability. Surprisingly, BAP mothers showed better implicit mentalizing but worse mental health than non-BAP mothers, which indicates the heterogeneity in the broader autism phenotype and the need to promote BAP mothers’ psychological resilience. The findings underscore the importance of contexts for implicit mentalizing and the need to profile mentalizing and mental health in BAP parents.

## Introduction

### Implicit and explicit mentalizing in autism

Mentalizing (or theory of mind) is the ability to attribute mental states (e.g. belief, intention, desire) to the self and others to explain and predict behaviours^1–3^. It is thought to consist of two systems: explicit mentalizing allows for a deliberate consideration of mental states, which is cognitively demanding and operates in a slow, flexible and conscious way; while implicit mentalizing allows for the efficient processing of mental states in a fast, rigid and unconscious way^4^. This ability allows people to understand everyday social contexts, thus the integrity of mentalizing ability is crucial for the effectiveness of social communication and interaction^5^. The social difficulties in autism^6^ have been suggested to result from mentalizing difficulties^2,3,7,8^, highlighting the importance of better understanding mentalizing to aid in identifying autism, help design more appropriate supports, and improve the lives of autistic people and their families. However, although some autistic adults perform less well than their non-autistic counterparts^9,10^, many autistic children and adults with greater verbal ability can pass mentalizing tasks^11–14^. This may also relate to the type of mentalizing system that each task design taps into.

It has been suggested that some autistic people without language difficulties may acquire the capacity to explicitly mentalize through compensatory learning, but still struggle to spontaneously attribute mental states^15^. In explicit mentalizing tasks, participants are encouraged to deliberately reason about mental states by employing tasks that involve direct questioning and require verbal responses. Thus, apart from explicit mentalizing, these tasks could also rely on language^11^ and other cognitive abilities, such as executive functions^16^ and memory^17^. Implicit paradigms were developed to bypass this issue to reveal the ability to spontaneously and quickly reason about others’ mental states^18^, by using more objective measurements, like eye movements^19^ and reaction time^20^.

Among various implicit tasks, Southgate et al.’s^19^ non-verbal anticipatory-looking paradigm was designed to detect more subtle false-belief reasoning than traditional explicit and some other implicit paradigms^19,21^. Senju et al.^22^ provided the first evidence for a dissociation between implicit and explicit mentalizing in autism by adapting Southgate et al.’s^19^ paradigm. Through tracking looking behaviour, Senju et al.^22^ found that autistic adults could not accurately predict actions on the basis of mental states, indicating that they were not spontaneously mentalizing about false beliefs, despite performing comparably to their non-autistic counterparts on explicit mentalizing tasks. Presumably, autistic adults may ‘hack’ the solution in explicit mentalizing tasks through compensatory strategies, such as linguistic abilities or executive functions^11,15–17,23–25^.

However, although studies with similar paradigms found that autistic children and adults have difficulties with implicit mentalizing but not explicit mentalizing^26–28^, this promising finding has been challenged in terms of the reliability of the paradigm^29,30^.

### The challenge of paradigm replication studies

Substantial evidence supports the idea that the anticipatory-looking paradigm can reliably detect implicit mentalizing in adults^26,31–33^. However, a considerable number of infant studies have not replicated Southgate et al.’s^19^ finding that 2-year-old non-autistic children can spontaneously appreciate others’ false beliefs and have argued that this paradigm should not be used with infants^32,34,35^.

Moreover, Kulke and colleagues conducted a series of replication studies to detect implicit mentalizing in children and adults, by closely following Southgate et al.’s^19^ paradigm, which involved two subtly different but conceptually similar false-belief trial types. Kulke et al.^36^ replicated the false-belief 1 condition in all age groups, but the false-belief 2 only in young adults; Kulke et al.^37^ replicated the false-belief 1, but not false-belief 2, condition; none of the other studies successfully detected implicit mentalizing in children or adults^29,38^. Accordingly, they suggested that there might not be spontaneous/implicit mentalizing, or that it exists but is hard to detect by anticipatory-looking paradigms, together with some other replication attempts^26,32,39^.

Three specific challenges have been made about the reliability of Southgate et al.’s^19^ paradigm, and several studies have endeavoured to overcome them. First, the single-trial design escalates error variance and dropout rate, which attenuate reliability^29,40^. A multi-trial design can improve the signal-to-noise ratio and increase power, allowing for a better estimation of individual performance. With such a design, Schneider et al.^33^ found that implicit mentalizing can be sustained over the course of a multi-trial procedure. Second, there is no matched true-belief condition, in which the observer’s and the agent’s beliefs should be consistent. However, the results from studies implementing true-belief conditions are mixed. It has been found that non-autistic infants and adults were able to attribute both true beliefs and false beliefs with low cognitive demands^39,41^; but, with the same paradigm, Kulke et al.^36^ did not find positive correlations between the two in any age group. Gliga et al.^42^ used a familiarization trial in Southgate et al.’s^19^ paradigm as a true-belief condition and concluded that siblings of autistic children were able to attribute others’ true beliefs, but not false beliefs.

Third, the paradigm might not be sufficiently engaging to elicit implicit mentalizing which is intrinsically a social ability^32,38,43,44^. Indeed, half of children in Southgate et al.^19^, 35–50% of adults in Kulke et al.^38^, and 70% of data in Schneider et al.^28^ were excluded due to failure to predict actions. Thus, Kulke et al.^38^ called for creating more engaging implicit paradigms to encourage mentalizing. To make Southgate et al.’s^19^ paradigm more engaging for children, Kulke and Rakoczy^45^ added verbal narrations of the events to the original non-verbal videos, and Kulke and Hinrichs^43^ moved the entire task to a more realistic social scenario; however, none of them replicated the original findings.

Although the replication was unsuccessful, Kulke and Hinrichs^43^ argued that when observers know there would not be any social consequence if they do not anticipate the agent’s action, reasoning about her mental state is less likely to be prioritized. The importance of social context is consistent with Woo et al.’s^44^ suggestion that socially evaluative contexts can facilitate mentalizing, defined as contexts where agents’ actions have interactive potential, including both prosocial and antisocial. They further proposed that the mixed results in replications using Southgate et al.’s^19^ paradigm may be because those studies have only detected false-belief reasoning within non-evaluative contexts, which provide observers less reason to care about agents’ mental states, as their actions are irrelevant. Thus, it is necessary to develop a more evaluative implicit mentalizing paradigm to evaluate whether social contexts can facilitate mentalizing.

### Broader autism phenotype (BAP)

The broader autism phenotype (BAP) was proposed to indicate a collection of sub-clinical expressions of autistic traits^46–50^. The BAP is qualitatively similar to autism, but neither leads to the full autism phenotype nor results in significant difficulties in socio-cognitive functioning^46,47^. Studies have observed that BAP is especially prevalent in the relatives of autistic people, for example, 20–40% of first-degree relatives but 2–9% of the general population^47^, indicating that autism is highly heritable^51–53^. Parents of autistic children (BAP) are about three times more likely to have autistic traits than parents of non-autistic children (non-BAP), especially in the communication and social skills domains^54–56^. Importantly, autistic traits in BAP parents are extremely heterogeneous: Rubenstein and Chawla^57^ found great variation in prevalence rates of the BAP across studies, ranging from 2.6 to 80%^48,57,58^.

BAP populations have been found to have similar social cognition challenges as autistic people^42,47,59^. Relatives of autistic people have moderate difficulties in mentalizing compared to non-autistic and autistic people^42,47^, and people with higher self-reported autistic traits show more difficulties in mentalizing^60^. Moreover, Livingston et al.^61^ found that BAP co-twins have mentalizing difficulties but can compensate for them at a behavioural level, which may potentially cause missed or late diagnosis^62^. Interestingly, compensation at the behavioural level, which may potentially cause missed or late diagnosis, has been observed more in autistic females than males^63–66^. It is possible that genetically predisposed individuals and those who are more likely to compensate have therefore been excluded from both the mentalizing and compensation literature because they do not meet the diagnostic criteria under the current clinical approaches^23^. Thus, implicit mentalizing and how its difficulties might be compensated in BAP populations, and BAP females in particular, have yet to be fully understood^24,47^. It is essential to explore BAP females’ socio-cognitive functioning^47,50^, which could, in turn, improve understanding of the endophenotypes of autism^53,67,68^.

One way to identify BAP females is as the mothers of autistic children. BAP mothers are also more vulnerable to mental health problems, such as depression and anxiety, compared with non-BAP mothers^69–71^. First, parenting and caring for an autistic child can be stressful^70,72,73^. Second, given that levels of autistic traits are associated with mental health outcomes, the elevated prevalence of autistic traits in BAP mothers may increase mental health problems^48,74–78^. Third, if BAP mothers engage in greater compensation, the heightened mental health problems in BAP relatives may result from the cost of compensation^24^. Given mothers’ primary role in parenting, it is vital to examine the relationship between BAP characteristics and mental health in mothers.

### The current study

The primary aim of the current study was to develop a more evaluative paradigm that provides more reason for eliciting mentalizing. To make Southgate et al.’s^19^ paradigm more evaluative, a question was added, prompting observers to anticipate agents’ actions. It might be argued that the prompt question might transform the task into an explicit task. Notably, only action anticipation, but not mentalizing, was prompted, to keep the paradigm implicit. Moreover, since eye-tracking has been considered as an applied implicit evaluation technique and widely used in autism research^22,79^, eye gaze as the outcome measure is implicit. Thus, the task did not make or require any explicit statement about mentalizing^45^. So far, more versus less socially evaluative contexts have not been compared directly in any replications^44^, thus we also include a comparable non-prompt version. According to Woo et al.’s^44^ proposal, the prompt implicit mentalizing task should enhance mentalizing compared with the non-prompt version. Additionally, we set out to employ a multi-trial design and include matched true-belief conditions to improve task reliability and replicability. This prompt paradigm would be evaluated in a sample of non-autistic young adults, and compared with a comparable non-prompted version to examine its potential to facilitate implicit mentalizing. According to Woo et al.^44^, we hypothesized that the prompt task would be better at enhancing belief reasoning than the non-prompt version.

By using the prompt task, our second aim was to identify the differences between BAP and non-BAP mothers in implicit and explicit mentalizing abilities, autistic traits, compensatory tendencies and mental health outcomes. According to the existing literature, we predicted BAP mothers would perform less well in mentalizing tasks, and reported more autistic traits, compensatory tendencies and mental health problems than non-BAP mothers. Last but not least, we aimed to explore how the aforementioned factors might relate to and predict implicit mentalizing performance in a non-clinical sample with sufficient statistical power.

## Method

### Participants

Two samples, a total of 128 participants, were recruited. In the *traits sample*, 68 participants from a local participant database were tested, aged 18–38 years (see demographics in Tables 1, 2). Five participants were excluded because of poor data quality (see *Data pre-processing* below), and two who reported an autism diagnosis were excluded from data analyses. Given the majority of the sample was college students, we reasonably assumed that they had average-to-high IQs which therefore was not tested.

**Table 1:** Descriptive statistics of the traits sample, mean (standard deviation).

	Traits (*n* = 61)
Age	21.97 (4.97)
Gender	Females (67.2%)
	Males (32.8%)
Handedness	Right (91.8%)
	Left (8.2%)
Education	High school (16.4%)
	UG (47.5%)
	PG (36.1%)
Anxiety (STAI Y-2)	39.27 (12.45)
Depression (BDI)	8.40 (9.36)
Autistic traits (AQ)	17.72 (7.66)
Autistic traits (BAPQ)	2.80 (0.70)
Camouflaging (CAT-Q)	3.40 (0.93)

*STAI Y-2* Spielberger state-trait anxiety inventory form Y-2, *BDI* beck depression inventory, *AQ* autism-spectrum quotient, *BAPQ* broad autism phenotype questionnaire, *CAT*-*Q* camouflaging autistic traits questionnaire, *UG* undergraduate, *PG* postgraduate.

**Table 2:** Descriptive statistics of the mothers sample, mean (standard deviation).

	BAP (*n* = 33)	Non-BAP (*n* = 26)
Age	42.55 (7.62)	41.73 (6.97)
Handedness	Right (87.9%)	Right (88.46%)
	Left (12.1%)	Left (11.54%)
Education	High school (21.2%)	High school (23.1%)
	UG (48.5%)	UG (34.6%)
	PG (30.3%)	PG (42.3%)
IQ (WASI-II) with range	107.09 (12.82): 81–132	106.23 (13.21): 72–133
Anxiety (STAI Y-2)	44.41 (10.36)	39.66 (7.92)
Depression (BDI)	13.11 (9.09)	8.12 (6.23)
Autistic traits (AQ)	16.94 (8.28)	15.81 (6.36)
Autistic traits (BAPQ)	2.93 (0.92)	2.70 (0.59)
Camouflaging (CAT-Q)	3.08 (1.17)	2.75 (0.84)

*WASI-II* Wechsler abbreviated scale of intelligence, second edition, *STAI* Y-*2* Spielberger state-trait anxiety inventory form Y-2, *BDI* beck depression inventory, *AQ* autism-spectrum quotient, *BAPQ* broad autism phenotype questionnaire, *CAT*-*Q* camouflaging autistic traits questionnaire, *UG* undergraduate; *PG* postgraduate.

In the *mother sample*, 60 participants took part but one was excluded from the analysis due to poor data quality (see “Data pre-processing” below), leaving 33 mothers of autistic children (BAP mothers), aged 20–57 years, and 26 mothers of non-autistic children (non-BAP mothers), aged 28–60 years. They were recruited through autism support groups in London, and advertisements placed around the local community. All participants in the BAP group stated that at least one of their children has an autism diagnosis from a qualified clinician but not themselves. None of the non-BAP mothers reported or was known to have a diagnosis of psychiatric or neurodevelopmental conditions or related family history. To avoid confounding variables, the two groups were required to be matched on age, handedness, highest education, and IQ as measured by the Wechsler Abbreviated Scale of Intelligence, Second Edition (WASI-II)^80^ (see Tables 2, 3).

**Table 3:** Group-wise comparison between the BAP and non-BAP groups.

	Inferential statistic, BAP (*n* = 33) vs non-BAP (*n* = 26)
Age	*t*(57) = 0.42, *p* = 0.674, *d* = 0.11
Handedness	χ^2^(1) = 0.005, *p* = 0.945
Education	χ^2^(2) = 1.27, *p* = 0.529
IQ (WASI-II) with range	*t*(57) = 0.25, *p* = 0.802, *d* = 0.07
**Anxiety (STAI Y-2)**	***t*(57) = 1.93, *p* = 0.059, *d* = 0.51 (marginal)**
**Depression (BDI)**	***t*(57) = 2.39, *p* = 0.020, *d* = 0.63**
Autistic traits (AQ)	*t*(57) = 0.25, *p* = 0.802, *d* = 0.15
Autistic traits (BAPQ)	*t*(55.04) = 1.16, *p* = 0.250, *d* = 0.29
Camouflaging (CAT-Q)	*t*(56.61) = 1.28, *p* = 0.206, *d* = 0.32

*WASI-II* Wechsler abbreviated scale of intelligence, second edition, STAI Y-2 Spielberger state-trait anxiety inventory form Y-2, *BDI* beck depression inventory, *AQ* autism-spectrum quotient, *BAPQ* broad autism phenotype questionnaire, *CAT*-*Q* Camouflaging autistic traits questionnaire.

Significant values are in bold.

Participants in both samples were required to be fluent in English and have normal or corrected-to-normal vision and hearing. Ethical approval for the study was received from the UCL Research Ethics Committee and all methods were performed in accordance with the approved guidelines and regulations. All participants gave written informed consent and were reimbursed for their time and effort.

### Procedure

Participants started the session by completing a demographic questionnaire, then a non-prompt implicit mentalizing task and a prompt version of the task, followed by the WASI-II (not for the *traits sample*) and an explicit mentalizing task. The session finished with a series of questionnaires measuring individual differences in autistic traits, camouflaging behaviour, anxiety, and depression. Participants were then fully debriefed. The overall duration of the experiment was 2 hours. One participant’s non-prompt task data in the *traits sample* were excluded as they did the prompt task before the non-prompt task. Testing was conducted either in participants’ homes or in the Institute of Cognitive Neuroscience, University College London.

### Implicit mentalizing tasks

The implicit mentalizing tasks were adapted from the anticipatory-looking paradigm in Senju et al.^22^, based on Southgate et al.’s^19^ classic false-belief task. One debriefing question was administered after the non-prompt and prompt tasks to investigate whether participants were aware of any differences between the two tasks.

#### Prompt task

**Figure 1:**
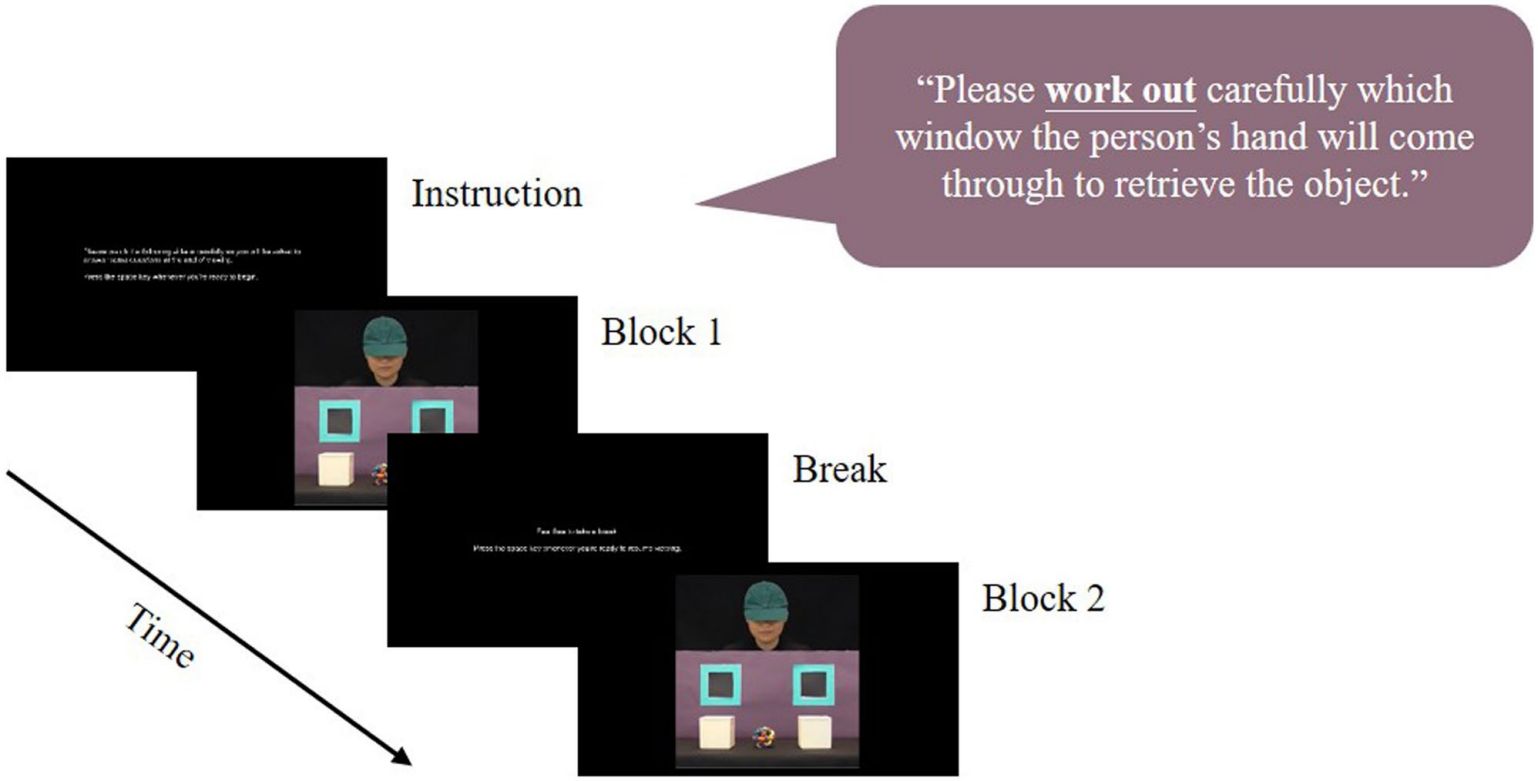
Prompt implicit mentalizing task procedure.

**Figure 2:**
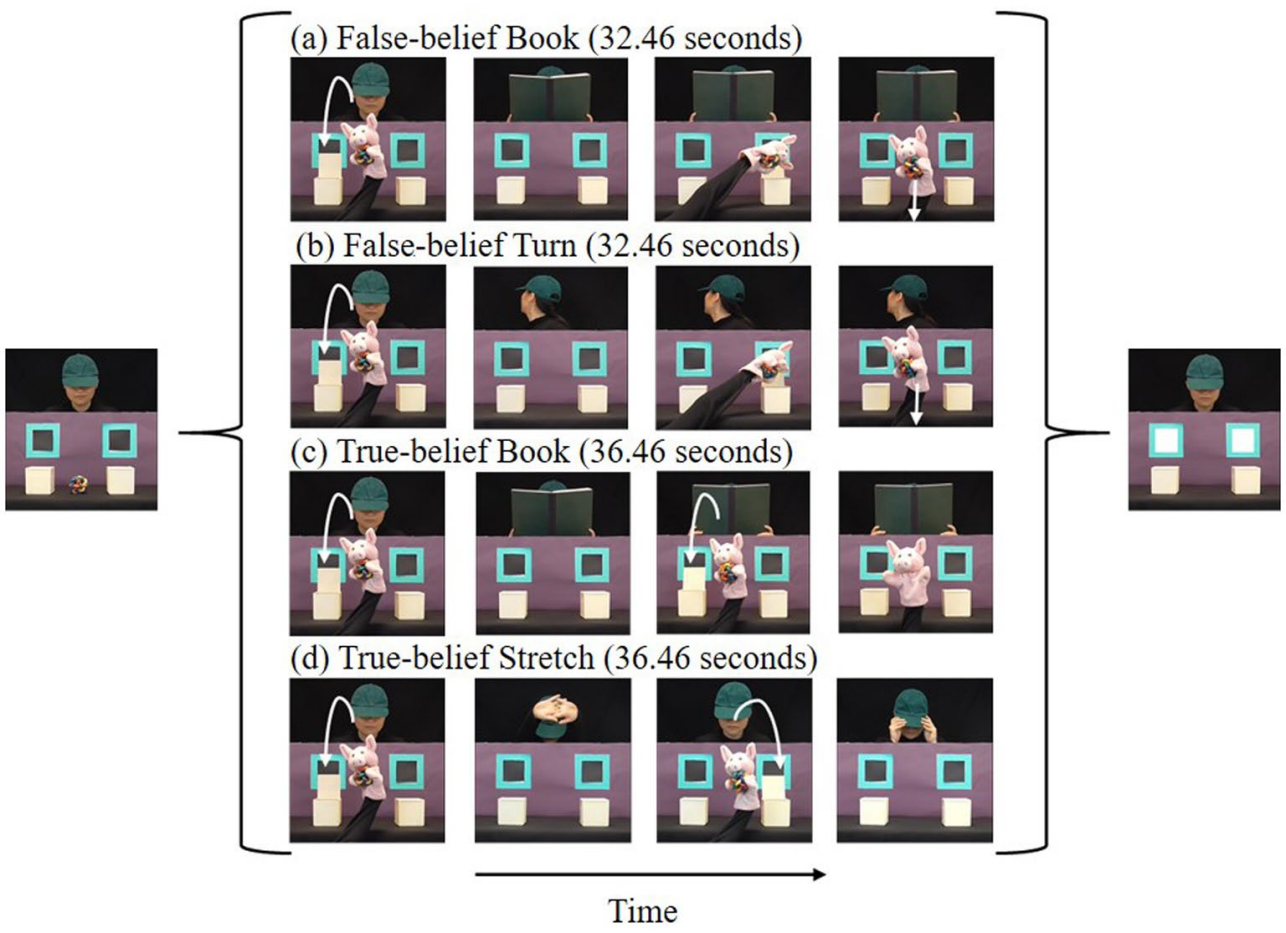
Selected key frames from the videos. (**a**) False-belief Book condition; (**b**) False-belief Turn condition; (**c**) True-belief Book condition; (**d**) True-belief Stretch condition.

Participants were prompted to reason about the agent’s mental state by asking them to predict her behaviour (see Fig. 1). In order to accurately predict the behaviour, they needed to be able to mentalize the agent’s belief. Participants were instructed to work it out in their minds, not answer out loud. There were 2 types of false-belief conditions and 2 types of matched true-belief conditions (see Fig. 2). The false-belief conditions included a Book condition in which the puppet removed the object from the scene while the agent was reading a book, and a Turn condition in which the puppet removed the object from the scene while the agent was distracted by the doorbell. Thus, observers should have different beliefs of the object’s location in false-belief conditions than the agent. The corresponding matched true-belief conditions included a Book condition in which the puppet moved the object out of a box and then back to the same box while the agent was reading a book, and a Stretch condition in which the agent came back to watch the scene after a quick stretching. Accordingly, both observers and the agent should have the same belief of the object’s location in true-belief conditions. The agent’s head always followed the puppet’s movement when she could see it, to indicate her attention.

The prompt task contained 2 experimental blocks (see Fig. 1). Each block had 2 trials of each of the 4 conditions. All participants watched the same pseudorandomized sequence of the trials to reduce inter-individual variability. The box where the puppet put the object, the hand that held the puppet and the side the agent’s head turned to were counterbalanced across the videos. An eye tracker was used to measure whether participants can predict which window the agent would open to retrieve the object by making anticipatory eye movements. If participants are mentalizing, they should look at the window/box which is consistent with the agent’s belief about the location of the object (*belief-congruent*).

This task was 15 min long with 1 break. Eye movements were recorded. Two questions were asked at the end of the task to encourage concentration. The questions asked about basic features of the videos (e.g. the colour of the puppet) and participants’ judgements (e.g. the most frequent final location of the object), but participants were not informed of the style of question in advance to avoid directing their attention to particular features of the videos.

#### Non-prompt task

**Figure 3:**
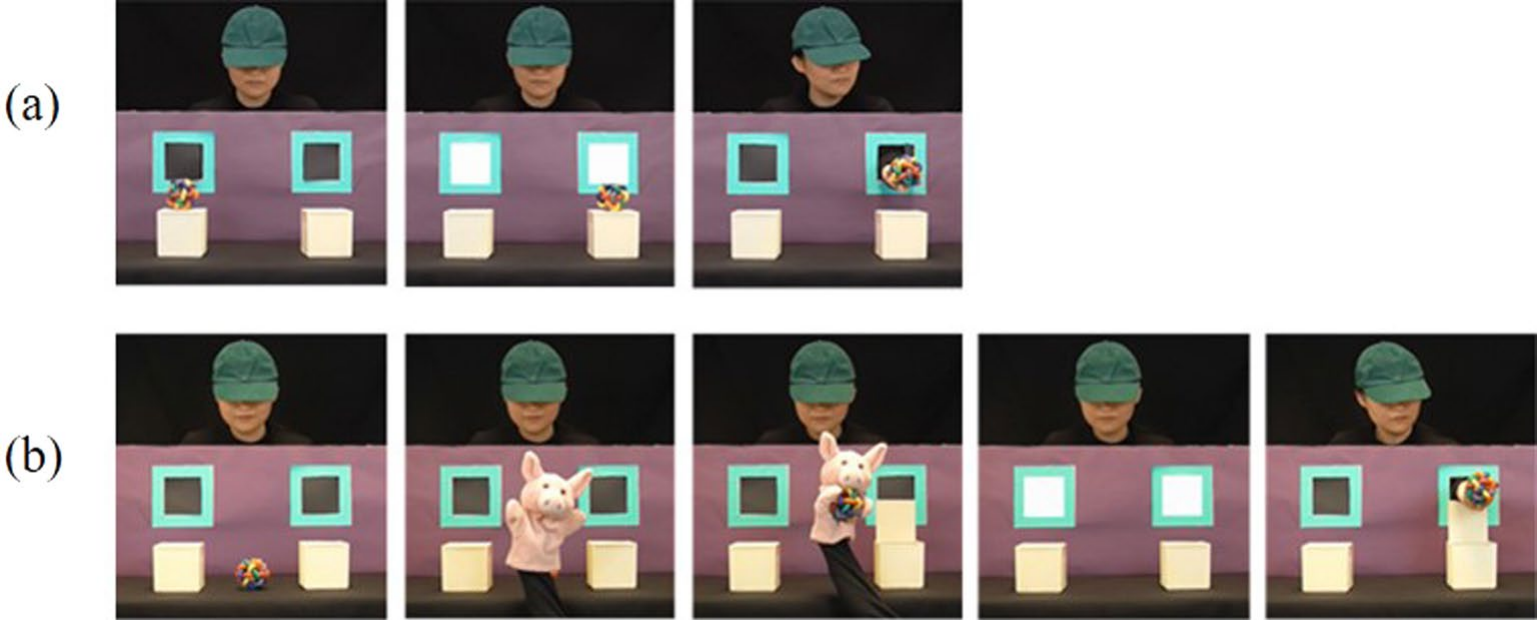
Familiarization trials. (**a**) Short trials; (**b**) Long trials.

The same stimuli were presented in the non-prompt task but participants were instructed to passively view the videos and answer some questions accordingly at the end. There was 1 familiarization block as well as 2 experimental blocks. The familiarization block enabled participants to implicitly learn the contingency that the agent was going to retrieve the object after the windows illuminated, which included 4 short and 4 long familiarization trials (see Fig. 3). Specifically, in the short trials, the object was on one of the boxes, and then the agent’s hand came through the window to retrieve it after the windows illuminated; while in the long trials, the puppet put the object into one of the boxes, and then the agent’s hand came through the window to open the box and retrieve it after the windows illuminated.

Each experimental block also started with 1 short and 1 long familiarization trials, followed by the 2 trials of each of the 4 conditions. The task was 20 min long with 2 breaks. At the end of this task, to encourage the participant to concentrate, two questions were asked about the details in the videos; to check that this task examined implicit processing, an 8-item funnelled debriefing procedure, adapted from Schneider et al.^81^, was administered.

#### Apparatus

A remote screen-based Tobii Pro X3-120 eye-tracker system, with a sampling rate at 120 Hz, was used to record eye movements (Tobii, Sweden). Visual and auditory stimuli were presented via a Dell Precision 5520 laptop (15.6-inch) with Tobii Pro Studio 3.4.8 software, integrated with the eye-tracker. Participants sat approximately 70 cm from the eye-tracker and were instructed to sit still throughout the eye-tracking assessment. A 5-point calibration was performed before each implicit task.

#### Areas of interest

**Figure 4:**
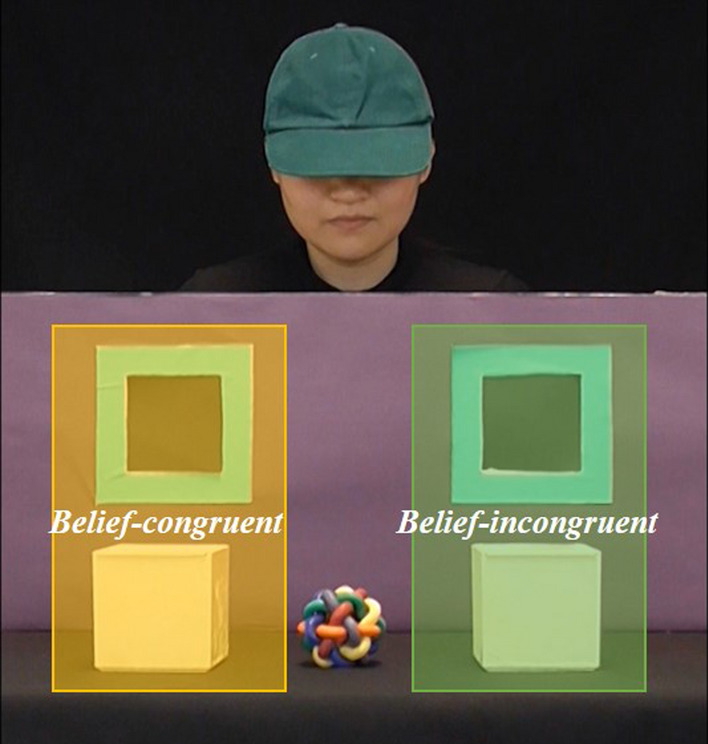
An example of the areas of interest: Belief-congruent (yellow) and Belief-incongruent (green).

Data were coded from the windows illumination onset to the end of each video, with a total duration of 5 s in each trial. Two areas of interest (AOIs) were identified: *Belief-congruent* and *Belief-incongruent* (see Fig. 4 as an example). Gaze data were extracted from both AOIs.

#### Fixation analysis

Data points with angular velocity below 30 degrees per second were classified as *fixations* (i.e. the visual gaze on a single location) while those above were *saccades* (i.e. the rapid eye movement between fixations). Two adjacent fixations with less than 75 ms time interval or less than 0.50 degrees visual angle were merged as one fixation. Fixations with less than 60 ms time duration were discarded. The total fixation duration was extracted, measuring the sum of the fixation durations within each AOI, by using Tobii Studio.

#### Data pre-processing

Differential looking scores (DLS), which measure participants’ looking preference between two visual targets, were calculated by dividing the difference between the total fixation duration to the *Belief-congruent* and *Belief-incongruent* AOIs by the sum of the two. DLS ranged from 1 to -1: closer to 1 if participants showed a looking bias towards the *Belief-congruent* AOI, closer to -1 if they were biased towards the *Belief-incongruent* AOI, and closer to 0 if they looked equally to both AOIs, equivalent to chance performance.

Three exclusion criteria were applied to ensure participants were paying attention to the task and the key events in the videos (e.g. watching the hand retrieving the object in the familiarisation trials). First, participants’ data from a task were excluded if they missed more than 25% data of that task. Second, the data from the non-prompt task were excluded for any participant whose average DLS in the familiarization block was missing or below chance, to confirm that they had paid attention to the key event (a combination to the prediction and the action itself). Third, the data from each experimental block of the non-prompt task were excluded if the average DLS of the two familiarization trials at the beginning of that block was missing or below chance. Accordingly, five *traits* participants and one BAP mother were excluded from the whole analysis.

### Explicit mentalizing task

The Strange Stories task is an advanced mentalizing test assessing participants’ ability to explicitly infer both *Mental States* and *Physical States*^10^. In this study, only the 8 *Mental States Stories* were used; accuracy scores therefore ranged from 0 to 16.

### Self-reported measures

Autistic traits were measured by the widely used Autism-Spectrum Quotient (AQ)^82^ and the Broad Autism Phenotype Questionnaire (BAPQ)^83^, with higher scores indicating more autistic traits. The AQ ranges between 0 and 50, Cronbach’s α = 0.90; the BAPQ between 1 and 6, α = 0.94. The BAPQ was also employed as it was specifically designed in a sample of BAP parents^83^, and showed superior internal consistency when compared with the AQ^50^. Social camouflaging (or compensatory) behaviours were measured by the Camouflaging Autistic Traits Questionnaire (CAT-Q)^84^, with higher scores indicating more strategies employed to cope with autistic characteristics during social interactions, ranging between 1 and 7, α = 0.92.

Anxiety traits were measured by the Spielberger state-trait anxiety inventory form Y-2 (STAI Y-2)^85^, with higher scores corresponding to more severe anxiety traits, ranging between 20 and 80, α = 0.92. Depression was measured by the beck depression inventory (BDI)^86^, with higher scores indicating more severe depressive symptoms, ranging between 0 and 63, α = 0.90. Item 9 regarding Suicidal thoughts was removed for ethical reasons. Missing values (item *n* = 16, with the number of missing responses less than 25% of the total number of items on each of these measures) were imputed using the individual’s mean scores of the scale or the sub-scale.

## Results

All effects are reported as significant at *p* < 0.05, and two-tailed *p* values were reported throughout, if not specified. Statistical analyses were conducted using IBM SPSS Statistics (Version 29).

### Validity of implicit mentalizing tasks

**Figure 5:**
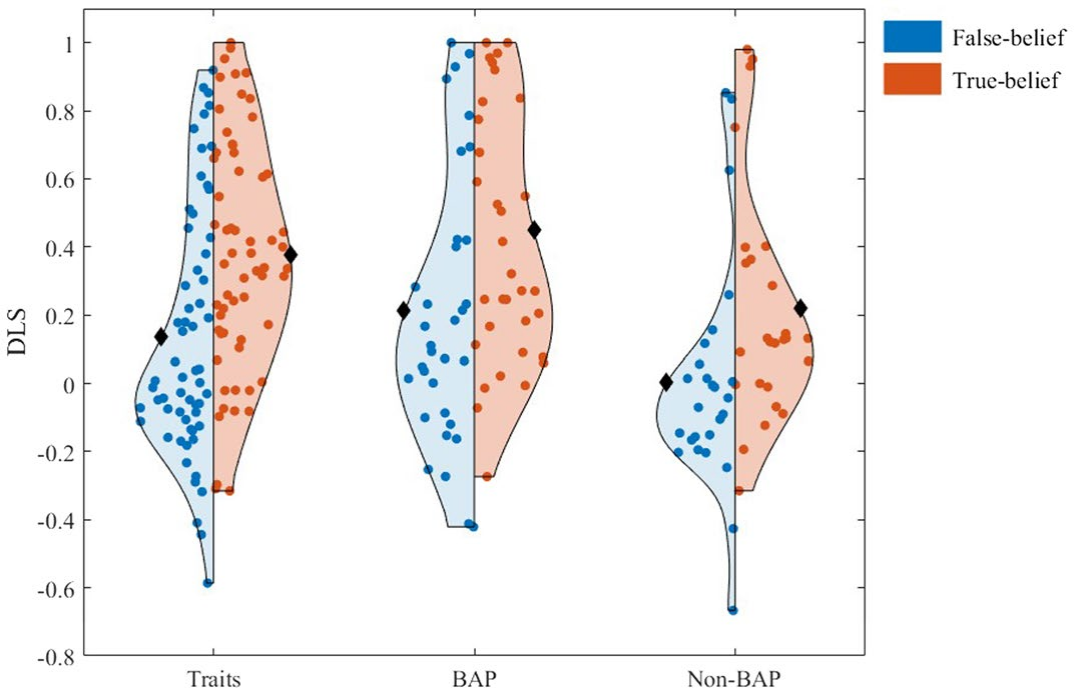
False-belief and True-belief DLS of the prompt task in the *traits* and *mother samples* (each dot represents the score of each participant); black diamonds represent the mean of each condition.

One-sample *t*-tests were conducted on the false-belief and true-belief DLS of both implicit tasks in the *traits sample*. The results showed that both false-belief and true-belief DLS were significantly above zero in the prompt task: false-belief: *t*(60) = 2.96, *p* = 0.004, *d* = 0.38, true-belief: *t*(60) = 8.65, *p* < 0.001, *d* = 1.11 (see Fig. 5). However, in the non-prompt task, only the DLS for the true-belief condition, but not for the false-belief condition, was significantly above chance: false-belief (*M* = − 0.05, *SD* = 0.25): *t*(59) = − 1.44, *p* = 0.154, *d* = − 0.19, true-belief (*M* = 0.09, *SD* = 0.27): *t*(59) = 2.64, *p* = 0.011, *d* = 0.34. Since the non-prompt task therefore showed poor validity, all non-prompt data were excluded in the following analyses. A paired samples *t*-test on the false-belief and true-belief DLS of the prompt task revealed that the performance in the true-belief condition was significantly better than the false-belief in the *traits sample*, *t*(60) = 4.79, *p* < 0.001, *d* = 0.89 (see Fig. 5).

### Comparing the mother groups

Self-report measure: As expected, compared with non-BAP mothers, BAP mothers scored significantly higher in anxiety (marginal) and depression, but unexpectedly not in autistic traits and camouflaging behaviours (see Tables 2, 3).

Implicit mentalizing: A two-way mixed-design analysis of variance (ANOVA) was conducted using the DLS as the outcome variable, Belief (false-belief, true-belief) as a within-subjects factor, and Group (BAP, Non-BAP) as a between-subjects variable. There were significant main effects of Belief, *F*(1, 57) = 29.88, *p* < 0.001, *partial η*^2^ = 0.344, and Group, *F*(1, 57) = 5.23, *p* = 0.026, *partial η*^2^ = 0.084, but no interaction. Similar to the *traits sample*, the true-belief condition had a higher DLS than the false-belief condition, but interestingly, BAP mothers scored higher than non-BAP mothers (see Fig. 5).

Explicit mentalizing: An independent samples *t*-test revealed that performance on the Strange Stories task was comparable between the BAP (*M* = 12.72, *SD* = 2.49) and non-BAP (*M* = 13.15, *SD* = 1.80) groups, *t*(57) = − 0.73, *p* = 0.466, *d* = − 0.19.

### Relationships

Given all participants in the *traits* and *mother samples* did not have an autism diagnosis, we combined the two samples to achieve an ideal statistical power for correlation and regression analyses. As the false-belief and true-belief conditions in the prompt implicit mentalizing task had a moderate-to-strong positive correlation, *r* = 0.55, *p* < 0.001, and there was no interaction between Belief and Group in the *mother sample*, these two conditions were merged by calculating the mean of each participant for the following analyses.

#### Correlations

**Figure 6:**
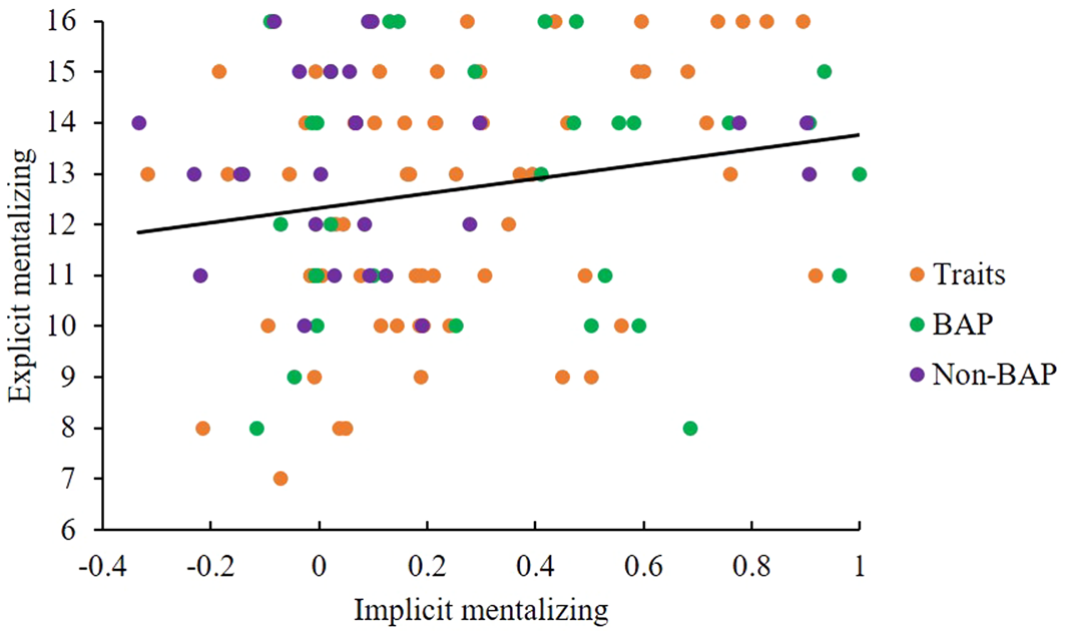
Correlation scatter plot between the DLS of the prompt implicit mentalizing task and the accuracy of the Strange Stories task measuring explicit mentalizing ability (each dot represents a participant).

**Figure 7:**
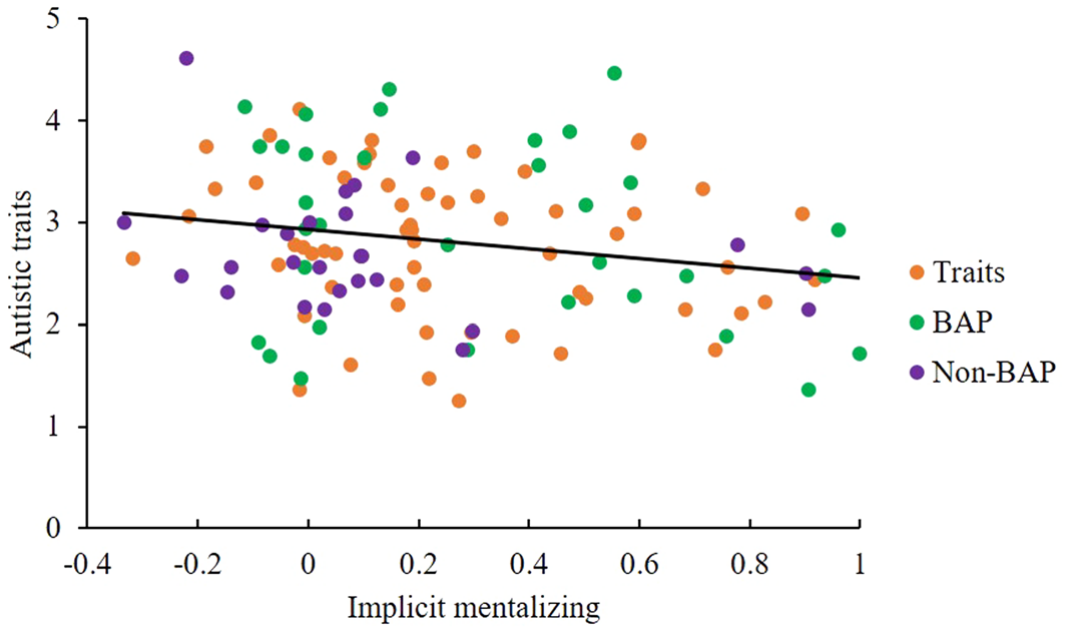
Correlation scatter plot between the DLS of the prompt implicit mentalizing task and autistic traits measured by the BAPQ (each dot represents a participant).

Correlations were investigated among the performance on implicit mentalizing (prompt task DLS), explicit mentalizing (strange stories task accuracy), individual differences in autistic traits (AQ, BAPQ), camouflaging (CAT-Q), anxiety (STAI Y-2) and depression (BDI), and age. Higher implicit mentalizing performance was significantly correlated with higher explicit mentalizing performance and with lower autistic traits (BAPQ) (see Figs. 6, 7 and Table 4). Age was positively related to depression (see Table 4). However, these relationships would not withstand correction for multicomparison. As expected, self-reported autistic traits (AQ, BAPQ), camouflaging, anxiety and depression were highly correlated with each other (see Table 4). A relationship between implicit mentalizing and autistic traits was observed with the BAPQ, but not the AQ; the former was therefore considered more sensitive in detecting autistic traits in a non-clinical population, in keeping with the existing literature^50,87^, and so the BAPQ was employed in the following regression analysis.

**Table 4:** Correlations (r) among the mentalizing performances, individual differences in autistic traits, camouflaging, mental health, and age.

	1	2	3	4	5	6	7	8
1 Implicit mentalizing (Prompt DLS)	–	0.20*	− 0.17	− 0.20*	− 0.004	− 0.04	− 0.10	− 0.10
2 Explicit mentalizing (Strange stories)		–	− 0.16	− 0.16	− 0.08	− 0.02	− 0.08	0.06
3 Autistic traits (AQ)			–	0.83***	0.56***	0.53***	0.45***	− 0.05
4 Autistic traits (BAPQ)				–	0.59***	0.62***	0.48***	0.03
5 Camouflaging (CAT-Q)					–	0.42***	0.29***	− 0.14
6 Anxiety (STAI Y-2)						–	0.68***	0.14
7 Depression (BDI)							–	0.20*
8 Age								–

*AQ* autism-spectrum quotient, *BAPQ* broad autism phenotype questionnaire, *CAT*-*Q* Camouflaging autistic traits questionnaire, *STAI* Y-*2* Spielberger state-trait anxiety inventory form Y-2, *BDI* beck depression inventory.

*p < 0.05, ***p < 0.001. Pearson’s correlation coefficients (*r*) are reported.

Significant values are in bold.

#### Regression

Multiple linear regression (enter method) was carried out with implicit mentalizing performance as the dependent variable and explicit mentalizing performance, age, autistic traits, camouflaging, anxiety, depression, and groups as potential predictors. Groups were coded as two dummy variables: BAP (BAP = 1, non-BAP & traits = 0), and non-BAP (non-BAP = 1, BAP & traits = 0), with *traits* as the reference category. The VIF values were below 3.89 and the tolerance statistics were above 0.26, which represents no multicollinearity. Results revealed that this model was significantly better at predicting the implicit DLS than using the mean of it, *F*(8, 111) = 2.80, *p* = 0.007,* R*^*2*^ = 0.17. The individual predictors were examined and showed that autistic traits and explicit mentalizing (marginal) were significant predictors of implicit mentalizing (see Table 5).

**Table 5:** Multiple linear regression model of predictors of the implicit mentalizing performance (the prompt DLS), n = 120.

	*b*	*SE b*	*β*	*t*(112)	*p*
Constant	0.196	0.238		0.824	0.412
**Explicit mentalizing (strange stories)**	**0.023**	**0.012**	**0.166**	**1.862**	**0.065 (marginal)**
Age	− 0.005	0.005	− 0.196	− 1.148	0.253
**Autistic traits (BAPQ)**	− **0.139**	**0.055**	− **0.323**	− **2.521**	**0.013**
Camouflaging (CAT-Q)	0.045	0.036	0.142	1.241	0.217
Anxiety (STAI Y-2)	0.004	0.004	0.145	1.077	0.284
Depression (BDI)	− 0.004	0.004	− 0.103	− 0.846	0.399
Group BAP	0.185	0.117	0.259	1.576	0.118
Group non-BAP	− 0.046	0.120	− 0.060	− 0.386	0.701

*R*^2^ = 0.15. *b* Unstandardized B, *SE b* coefficients standard error, *β* standardized coefficients beta. *BAPQ* broad autism phenotype questionnaire, *CAT*-*Q* Camouflaging autistic traits questionnaire, *STAI Y*-*2* Spielberger state-trait anxiety inventory form Y-2, *BDI* beck depression inventory.

Significant values are in bold.

## Discussion

The current study aimed to develop a more evaluative implicit mentalizing paradigm by implementing a prompt question, a multi-trial design and matched true-belief conditions to improve task reliability and replicability and assess it in a non-autistic young adult sample. We then explored the relationship between implicit and explicit mentalizing abilities, autistic traits, compensatory tendencies and mental health outcomes in a non-clinical sample with sufficient statistical power. Third, we compared the aforementioned abilities and characteristics between BAP and matched non-BAP mothers.

### Prompt task validation

Three main pieces of evidence indicate that the prompt implicit mentalizing task is valid, and may be better at facilitating mentalizing than the non-prompt version. First, both true-belief and false-belief conditions were performed significantly above chance in a group of non-autistic adults, meaning that participants showed a looking bias towards the *belief-congruent* AOI in this task, which conceptually replicated previous findings^26,31–33^. Accordingly, non-autistic people are able to predict the agent’s behaviour by implicitly reasoning about her mental states. This indicates that the task is able to facilitate mentalizing and elicit belief-based action prediction in the general population, supporting the prompt task as a valid implicit mentalizing task. On the other hand, the false-belief condition in the non-prompt version did not differ from chance. That is, participants did not show a preference for the *belief-congruent* location in the false-belief condition, which is consistent with some previous unsuccessful replications from Kulke and colleagues^29,36–38^. This suggests that the non-prompt task was unable to elicit false-belief reasoning, indicating that it might not be a reliable paradigm.

In line with our hypothesis, this preliminary evidence seems to suggest that the more evaluative prompted task is indeed better at facilitating mentalizing than the less evaluative non-prompt task, which is consistent with Woo et al.’s^44^ proposal. However, as we created our own stimuli to conceptually replicate Southgate et al.’s^19^ paradigm, we cannot rule out the possibility that small changes in the non-prompt task resulted in its invalidity. One such deviation is that we removed the delay phase in the familiarization trials between the end of the audio-visual cue and the onset of the agent’s action, to make the task more realistic. Schuwerk et al.^32^ suggested that their unsuccessful replication might be because this phase was too long to build up the contingency between the cue and the action. Similarly, Kulke and Hinrichs^43^ reported adult participants noticed the artificial waiting time and suggested a shorter and more realistic delay should improve task reliability. Removing it altogether may have been too drastic, however, and a delay may in fact be needed to establish the contingency; future studies should modify the timing and further investigate the importance of context in mentalizing^44^. However, this also indicates that the non-prompt paradigm may be more fragile than the prompt version, needing more strict criteria to elicit mentalizing, which further confirms our primary hypothesis.

Second, the performances of explicit and implicit mentalizing were positively correlated, and, although borderline, the former can affect the latter to a degree, which is consistent with our prediction. This suggests that the two tasks may tap into overlapping cognitive mechanisms, confirming that the prompt implicit mentalizing task was valid to measure mentalizing. Although implicit mentalizing and explicit mentalizing are thought to work both complementarily and oppositionally^88^, EEG and fMRI studies have revealed that implicit and explicit mentalizing are elicited at about the same time and have a shared neural network, including the medial prefrontal cortex and the temporoparietal junction^89–91^. Given general cognitive factors, such as language, memory and attention likely influence explicit more than implicit mentalizing^4,11,16,17,42^, it is perhaps unsurprising that some studies have not shown relationships between implicit and explicit mentalizing performance^29,92,93^ when other factors play a more key role in particular tasks.

Third, we found that autistic traits were not only negatively associated with but also affected implicit mentalizing, which indicates that higher autistic traits may be a sign of poor implicit mentalizing in non-autistic populations. This result replicates previous observations of negative correlations between autistic traits and implicit mentalizing in both autistic^94^ and non-autistic populations^95^ and is consistent with the idea that autistic people may have specific difficulties in implicit mentalizing, while some develop relatively good explicit mentalizing later in deveopment^11,15–17,22–25,61^. Accordingly, we can more confidently state that the prompt task is able to authentically measure implicit mentalizing.

However, we did not replicate the relationship between autistic traits and explicit mentalizing previously reported in autistic people^60^. Also, neither type of mentalizing ability was correlated with compensatory tendencies, anxiety, depression and age in the entire sample, and none of these four factors could account for variance in implicit mentalizing performance. Thus, we did not replicate Livingston et al.’s^61^ finding in autism that weaker mentalizing and lower autistic traits are related to higher mental health problems because of compensation. Again, this might be because autism does not result from explicit mentalizing difficulties or lead directly to higher compensatory tendencies or mental health problems. Other factors might play more essential roles in the development of explicit mentalizing and compensatory tendencies, like executive function or language^11,16,61^, as well as mental health outcomes^23,24,63,84^. Together with the fact that autistic traits are relatively low in non-autistic populations, the lack of associations observed in our non-clinical sample is understandable.

It is also possible that self-reported inventories for assessing autistic traits, compensation and mental health might measure the awareness or the perceived social expectations of these characteristics instead of genuine individual differences^96^. Although self-reported questionnaires are the most common instruments, which are money- and time-saving, these measures may be influenced by the BAP^54,59,97^ and unconscious compensatory mechanisms^23,63^, thus, more objective measures are needed in future studies^61,75,83,98^.

We also replicated Surian and Geraci^41^ and Wang and Leslie^39^, but not Kulke et al.^36^, that true-belief attribution was positively correlated with false-belief attribution, and true-belief conditions were consistently performed better than false-belief conditions in all samples. This relationship has also been observed in neuroimaging studies. Nijhof et al.^99^ observed that the right temporoparietal junction was recruited in both true-belief and false-belief reasoning, and more so during false-belief than true-belief conditions, in both implicit and explicit mentalizing. Similarly, Schneider et al.^81^ found the same pattern in the superior temporal sulcus, but not in the rest of the mentalizing network. We can assume therefore that both true-belief and false-belief reasoning recruit mentalizing to a degree, but given the differences in accuracy in our task and differences in brain activation in the literature, false-belief reasoning requires higher mentalizing abilities than true-belief reasoning.

### BAP

Surprisingly, BAP mothers performed better in the implicit but comparably in the explicit mentalizing tasks compared with non-BAP mothers, which is not consistent with Gliga et al.’s^42^ study of infant BAP siblings. One potential explanation is that, because of a lack of group difference also in autistic traits, the BAP mothers in our sample did in fact have strong implicit mentalizing abilities. Unlike many infant siblings, it is possible that some or all BAP mothers do not possess autistic traits or autistic cognitive profiles, are not genetically predisposed to autism themselves and hence do not contribute to their child’s genetic predisposition.

However, the lack of group differences in autistic traits may not necessarily mean BAP and non-BAP mothers are indistinguishable. An et al.^53^ found that BAP mothers had smaller grey matter volumes in the right middle temporal gyrus, temporoparietal junction, cerebellum, and parahippocampal gyrus than non-BAP mothers, even when group differences in autistic traits were absent. This might suggest the presence of subtle underlying neurological differences despite a lack of autistic traits, or alternatively that our BAP mothers were not representative of the wider BAP mother population and were totally unaffected at the behavioural, cognitive and neurological level.

Alternatively, it might be that an interaction between protective factors and autistic advantages boosted BAP mothers’ performance in the prompt task. BAP parents are believed to reflect an underlying genetic liability for autism^54^, for example, the shared genetic overlap between BAP mothers and their autistic children has been observed to be associated with the mothers’ autistic traits^100^. Notably, autism is not only associated with social difficulties but also with remarkable skills and talents^101,102^, for example, a detail-focused cognitive style^102^. Together with the finding that females require more inherited factors than males to exhibit autism^103^, BAP mothers might possess some protective factors that mean they display fewer autistic traits than their children, but reserve some autism-like cognitive styles that predispose them to better develop certain cognitive abilities than non-BAP mothers^102^.

A third explanation is that the BAP mothers may have possessed higher motivation to engage in the task because of their autistic children, and therefore, performed better in the more passive implicit task. However, in the explicit task, engagement might not enhance performance, as the already highly evaluative context^44^ may mean participants are already fully engaged. Although Southgate et al.’s^19^ paradigm is well-known in the literature and presumably in autism communities, it is unlikely that the BAP group knew the task expectations beforehand, otherwise, they might have also performed well in the non-prompt version.

Although no group difference was found in self-reported autistic traits and compensatory tendencies, BAP mothers reported higher levels of depressive and marginally higher levels of anxious symptoms than non-BAP mothers. These results support the idea that the mental health difficulties in BAP mothers might be more related to their chronic stress from parenting and caring for autistic children^70,72,73,104^ than their own autistic traits^48,74–78^ or the cost of compensation^24^. However, the current study cannot rule out a multi-risk model of mental health outcomes in BAP mothers, as the BAP is highly heterogeneous in relatives of autistic people^55,57^. On all accounts, support is needed to alleviate mental health issues and develop psychological resilience in BAP mothers^72^.

In addition, positive correlations were reported among autistic traits, compensatory tendencies and mental health problems in the merged large sample. These findings are consistent with the extant literature that individuals with more socio-cognitive difficulties^47,82,83^ need to allocate more cognitive resources to compensate for their core difficulties, which is likely to compromise their mental health in both autistic and non-autistic populations^23,24,61,63,105^.

### Advantages & limitations

One advantage of the current study is the use of a prompt question in the implicit mentalizing task. This adaptation seemed to increase the task context evaluative-ness, which makes the prompt anticipatory paradigm more robust in facilitating implicit mentalizing and therefore improves the task reliability and replicability^43,44^. However, a corresponding limitation of our task design is that the non-prompt and prompt task order could not be counterbalanced. If the prompt task was performed first, the non-prompt task would logically become a prompt version. Nevertheless, it seems unlikely that the fixed procedure can account for our primary findings.

Another advantage lies in directing attention towards the BAP, an area that still holds significant gaps in understanding. This may not only have significant implications for autism research^53,67,68^ but better support families with autistic children^72^. Nonetheless, because of the female sample, our results cannot be generalized to the entire BAP community, particularly as recent studies have suggested that the BAP is more prevalent in BAP fathers than mothers^57,106^. Accordingly, the lack of group differences between our BAP and non-BAP mothers, especially in autistic traits, seems to imply that our BAP mothers did not have autistic characteristics. Future studies should include both parents to reveal patterns in the whole family and sex- and gender-informed phenotypes of autism^59,75,84,104,107^.

We acknowledge two additional limitations. The current study employed a cross-sectional design, so the direction of the association between mentalizing abilities, autistic traits, compensation and mental health cannot be conclusively determined. Future research should incorporate a longitudinal design to confirm the causality of these relationships. Furthermore, we had relatively small samples, especially the non-BAP sample, which may compromise the power to detect group differences. Future research would benefit from recruiting larger samples.

## Conclusion

In closing, the current study developed a more evaluative implicit mentalizing task which was proved to be robust in facilitating false-belief and true-belief reasoning^44^. With the adapted prompt task, we found that both explicit mentalizing and autistic traits are associated with implicit mentalizing but not with each other, which supports the idea of two distinct but overlapping mentalizing systems^4^ and implicit but not explicit mentalizing difficulties in autistic adults^15,22^. However, BAP mothers showed better implicit mentalizing and worse mental health than non-BAP mothers, but no other differences, which indicates the heterogeneity within the broader autism phenotype^55,57^ as well as the need to support families with autistic members in terms of mental health and psychological resilience^72^. Future studies are needed to further examine the prompt task reliability and validity and investigate associations among autism, mentalizing, compensation and mental health in more clinical and sub-clinical populations.

## Data Availability

The dataset supporting the conclusions of this article is available in the Open Science Framework repository, at https://osf.io/mznaw/. Materials in the current study are available on request from the corresponding authors.
